# One-minute sit-to-stand test is practical to assess and follow the muscle weakness in cystic fibrosis

**DOI:** 10.1186/s12931-022-02176-6

**Published:** 2022-09-23

**Authors:** Sophie Hardy, Silvia Berardis, Anne-Sophie Aubriot, Gregory Reychler, Sophie Gohy

**Affiliations:** 1 Pneumology Department, Cliniques Universitaires Saint-Luc, Avenue Hippocrate 10, 1200 Brussels, Belgium; 2 Pediatric Department, Cliniques Universitaires Saint-Luc, Brussels, Belgium; 3 Cystic Fibrosis Reference Center, Cliniques Universitaires Saint-Luc, Brussels, Belgium; 4 Pole of Pneumology, ENT and Dermatology, Université Catholique de Louvain (UCLouvain), Avenue Hippocrate 54/B1-54.04, 1200 Brussels, Belgium

**Keywords:** Cystic fibrosis, Quadriceps strength, Functional exercise capacity, One-minute sit-to-stand test, Intravenous antibiotherapy

## Abstract

**Background:**

Quadriceps muscle weakness and reduced exercise tolerance are prevalent and associated with a worse prognosis in patients with cystic fibrosis (CF). The one-minute sit-to-stand test (1STST) has been proposed to evaluate functional exercise capacity and quadriceps strength.

**Research question:**

The aim of the study was to verify the relationship between the 1STST and the maximal isometric voluntary contraction of the quadriceps (MVCQ) evaluated by the dynamometer in stable patients with CF and to evaluate the impact of intravenous (IV) antibiotherapy.

**Methods:**

Dynamometer and 1STST were performed in stable patients with CF at a routine visit, the admission and the discharge of an IV antibiotherapy. Patients wore an activity monitor during 72 h during IV treatment.

**Results and significance:**

51 stable patients with CF at a routine visit and 30 treated with IV antibiotherapy were recruited. In stable patients, the 1STST was reduced to a mean of 2101 nxkg (657—SD), representing a median of 79% (7; 142—min; max)) of the predicted values (%PV) as well as the MVCQ to 78.64 N-m (23.21; 170.34), representing 57%PV (26). The 1STST was correlated to MVCQ (r = 0.536; p < 0.0001) and lung function (r = 0.508; p = 0.0001). Over the IV antibiotherapy course, the 1STST improves significantly like lung function and body mass index while a positive trend for MVCQ was observed. The gain of 1STST was correlated to the change in MVCQ (r = 0.441; p = 0.02) and was significantly higher in hospitalized patients versus home therapy. The 1STST is a good alternative to the dynamometer to evaluate and assess muscular weakness for the routine visit and IV antibiotherapy.

**Supplementary Information:**

The online version contains supplementary material available at 10.1186/s12931-022-02176-6.

## Highlights

- The 1STST is a practical test to follow muscular weakness in patients with CF.
- The reduction of 1STST in patients with CF is correlated to MVCQ and to FEV1.
- The 1STST improved after an IV antibiotherapy, especially with rehabilitation.

**Supplementary Information:**

The online version contains supplementary material available at 10.1186/s12931-022-02176-6.

## Background

Quadriceps muscle weakness and reduced exercise tolerance are prevalent in cystic fibrosis (CF) and are independent factors of mortality [1–3]. They are associated with a worse prognosis, a reduced quality of life, an increased risk of exacerbation and hospitalization, and a more rapid lung function decline [1, 4–6].

Causal factors of muscle dysfunction (muscle atrophy, weakness and endurance) are intrinsic and extrinsic, worsening the evolution of the disease. The sedentary lifestyle and muscle immobilization negatively impact the quadriceps strength [3]. Moreover, as the dysfunctional chloride channel seems expressed in skeletal muscle, altered ionic homeostasis and abnormalities in ATPase and mitochondrial activities could contribute to impaired muscle’s contractility and fatigability [7, 8]. In addition, the ventilatory limitation influences negatively the exercise capacity [9] while exacerbations reinforce inflammation, sedentarity and denutrition, further decreasing muscle strength [7]. Indeed, the quadriceps strength in adults with CF at the hospital admission for an exacerbation, is lower than at discharge and one month after hospitalization (recovering to the same level at the admission) without any training program [10]. A reduced strength was also observed in children without training program at discharge [11]. The activity level of the patient is related to peripheral muscle strength during exacerbations [12]. The exercise training is one of the most important intervention against muscle weakness and reduced exercise tolerance [13].

The dynamometer is the commonest tool for muscular strength evaluation with the maximal isometric voluntary contraction of the quadriceps (MVCQ) [14]. The one-minute sit-to-stand test (1STST) contributes to assess both functional exercise capacity and quadriceps strength [15–18]. In COPD, it was shown to be reproducible, easy to perform and less demanding for the cardiorespiratory system than the cardiopulmonary exercise testing (CPET) [19]. In adult patients with CF, a correlation between the 1STST and the peak VO2 (CPET) as well as with quadriceps strength was shown [20–22]. The 1STST is a valid functional exercise capacity test similarly to the 6-min walking test [23].

Our hypothesis is that the 1STST can assess peripheral muscle weakness and exercise tolerance in cystic fibrosis during routine visit as well as during an intravenous (IV) antibiotherapy. Secondly, all hospitalized patients could better improve these parameters than outpatient, especially with a daily exercise training. The primary objective of our study was to evaluate the quadriceps muscle strength and functional exercise capacity in stable patients with CF, with the 1STST and the dynamometer. The secondary aim was to assess the impact of an acute or elective IV antibiotherapy, followed at home or in hospital, on the quadriceps force, exercise capacity and physical activity of the patients.

## Methods

### Study population

All patients with CF followed at the Cliniques Universitaires Saint-Luc were recruited between February 2017 and October 2019. The study was approved by the local ethic committee (2017/26JAN/046) and registered (NCT03117764). All participants signed a written informed consent.

Inclusion criteria were a diagnosis of CF following the definition of Rosenstein [24], to be in stable condition (primary objective), or to be treated by IV antibiotic for an exacerbation or electively (*Pseudomonas aeruginosa* eradication or precarious patient) (secondary objective). Pulmonary exacerbation was defined, according to European Consensus Group [25], as the need for additional antibiotic treatment based on a change in at least two of the following criteria: sputum volume or color, cough, malaise, fatigue or lethargy, anorexia or weight, pulmonary function by 10% or more, radiography, and dyspnea. The patients were treated at home or at hospital depending on the severity of the exacerbation and the compliance of the patient. Hospitalized patients followed a twice daily training program under the supervision of qualified physiotherapists, contrarily to the patients treated at home but all the patients (at home and hospitalized) received a daily supervised airway clearance by a chest physiotherapist.

Exclusions criteria were musculoskeletal conditions interfering with mobility or assessment of the muscle strength, pregnancy, or pulmonary graft. Advanced cystic fibrosis lung disease, enteral feeding and diabetes are not considered as exclusion criteria for exercise testing.

For each patient, age, body mass index (BMI), smoked history, microbiology, genotype, sweat chloride concentration and pancreatic sufficiency or insufficiency were retained. For patients with acute exacerbation, a blood test with C-reactive protein (CRP) and immunoglobulin G (IgG) (inflammatory markers) and a chest X-ray were also registered. A spirometry following ATS-ERS guidelines was performed at inclusion, at the first and the last days of the IV antibiotherapy.

### Outcomes

To evaluate muscular strength and exercise capacity, the 1STST and the Microfet2dynamometer®, (Hoggan, Biometrics, France) were prospectively used during a routine visit for the stable patients, and at the beginning and the end of an IV antibiotherapy.

The 1STST was performed, using a chair of 46 cm in height, without arm rests, as described by Strassmann et al. [26]. To reduce the learning effect, the test was first demonstrated by the operator, and subjects had the opportunity to perform a few practice cycles to ensure correct realization [21]. Patients must fully stand up (leg straight) and then sit down (buttocks on the chair, knees at 90° flexion), as many times as possible during one minute. They are allowed to rest if necessary and standardized encouragements were given. Reference values from Strassman were used and extrapolated for patients below 20 years old [26]. The 1STST was expressed in number of repetitions per minute as well as in product of the bodyweight (nxkg). The MVCQ was evaluated with the Microfet2 dynamometer as described previously with a hand-belt stabilization, on the dominant leg, during 6 consecutive efforts and with standardized encouragements [15, 27]. The level arm was measured in order to express the MVCQ in Newton-meter (N-m) and to compare it with the reference values from Hogrel [28]. The 1STST and MVCQ were measured during a routine visit for all patients. The 1STST and the MVCQ force were assessed in the first 24 h and on the last day of the IV antibiotherapy.

In addition, patients had to wear the accelerometer (ActiLife 6, ActiGraph Software, The Netherlands) from the first to the third day of the IV antibiotherapy, during day and night to estimate physical activity. The number of steps was recorded and expressed in the mean number of steps per 24 h.

### Statistical analysis

The sample size was determined. Forty-six subjects were required. Taking into account a 10% drop-out rate, the total sample size was set to 50 patients. Normality of the data was assessed with Komolgorov and Shapiro–Wilk tests. Descriptive results were shown by means and standard deviation for parametric data or medians and interquartile range or minimum–maximum for non-parametric data. Mann–Whitney U test (for 2 groups of unpaired non-parametric data), Student t-test (for 2 groups of minimum unpaired 20 parametric data), Wilcoxon test test (for 2 groups of paired non-parametric data), paired Student t-test (for 2 groups of minimum paired 20 parametric data), and Chi-square test (for unpaired data and the analysis of differences between 2 categorical variables) were used as appropriated. Correlation coefficients were determined by Pearson test if parametric and by Spearman test if non-parametric. A *p* value less than 0.05 was considered as statistically significant. Statistical analyses were performed using IBM SPSS Statistics (version 25 for Windows, Chicago, USA) and figures were done using GraphPad Prism (version 8.00 for Windows; GraphPad Software, San Diego, USA; www.graphpad.com).

## Results

### Study population

We recruited 51 stable patients with CF at a routine visit and 30 patients treated with IV antibiotherapy. All characteristics of the patients are summarized in Tables 1 and 2.

**Table 1:** Characteristics of the patients with CF in stable condition

Subjects, n	51
Sex (F/M)	14/37
Age, years	31 ± 14
Children (< 18 years)/Adults	9/42
F508del/F508del (yes/no)	25/26
Sweat Chloride, mmol/L	106 (29; 140)
Pancreatic sufficiency (yes/no)	14/37
*Pseudomonas aeruginosa* chronic infection (yes/no)	19/32
Diabetes (yes/no)	9/42
Smokers (yes/no)	4/47
BMI, kg/m^2^	21.05 (14.65; 33.80)
FEV1, L	2.69 ± 1.17
FEV1, %PV	74.21 ± 22.37
1STST, n	34 (17; 68)
1STST, %PV [26]	79 (7; 142)
1STST, nxkg	2101 ± 657
MVCQ, N-m	78.64 (23.21; 170.34)
MVCQ, %PV [28]	56.73 ± 26.21

Data are mean ± SD if parametric or median (min; max) if non-parametric data (Kolmogorov and Shapiro–Wilk tests). *Definition of abbreviations*: n, number; F, female; M, male; yrs, years; mmol/L, millimoles per liter; BMI, body mass index; kg/m^2^; kilogram divided by the square meter; FEV1, forced expiratory volume in one second; L, liter; PV, predicted values; 1STST, the one-minute sit-to-stand test; nxkg, number of repetitions as a product of bodyweight expressed in kilogram; MVCQ, maximal isometric voluntary contraction of the quadriceps; N-m, Newton-meter

**Table 2:** Characteristics of the patients with CF receiving IV antibiotics

Subjects, n	30
Sex (F/M)	12/18
Age, years	36 ± 14
Children (< 18 years)/Adults	5/25
F508del/F508del (yes/no)	14/16
Sweat Chloride, mmol/L	100.00 (23.40; 127.40)
Pancreatic sufficiency (yes/no)	10/20
*Pseudomonas aeruginosa* chronic infection (yes/no)	15/15
Diabetes (yes/no)	6/24
Smokers (yes/no)	3/27
BMI, kg/m^2^	20.51 (17.63; 31.09)
FEV1, L	1.98 ± 0.81
FEV1, %PV	56.79 ± 14.70
1STST, n	33 (18; 57)
1STST, %PV [26]	69.23 (40.91; 125.53)
1STST, nxkg	1976 (1035; 3477)
MVCQ, N-m	73.48 ± 31.34
MVCQ, %PV [28]	50.15 (23.86; 142.26)
CRP, mg/L (n = 29)	3 (1; 133)
IgG, g/L (n = 27)	10.50 (6.30; 25.10)
Chest X-Ray – new infiltrate (yes/no) (n = 13)	4/9
Exacerbation/Elective	11/19
Hospital/Home	16/14
Steps, n/24 h	8371.12 ± 4463.73

Data are mean ± SD if parametric or median (min; max) if non-parametric data (Kolmogorov and Shapiro–Wilk tests). N is specified when data are missing. *Definition of abbreviations*: n, number; F, female; M, male; yrs, years; mmol/L, millimoles per liter; BMI, body mass index; kg/m^2^; kilogram divided by the square meter; FEV1, forced expiratory volume in one second; L, liter; PV, predicted values; 1STST, the one-minute sit-to-stand test; nxkg, number of repetitions as a product of bodyweight expressed in kilogram; MVCQ, maximal isometric voluntary contraction of the quadriceps; N-m, Newton-meter; CRP, C-reactive protein; mg/L, milligram per liter; IgG, G-immunoglobulin; g/L, gram per liter; n/24 h number of steps per day

### Peripheral muscular strength and functional exercise capacity in children and adults with CF in stable condition

**Fig. 1:**
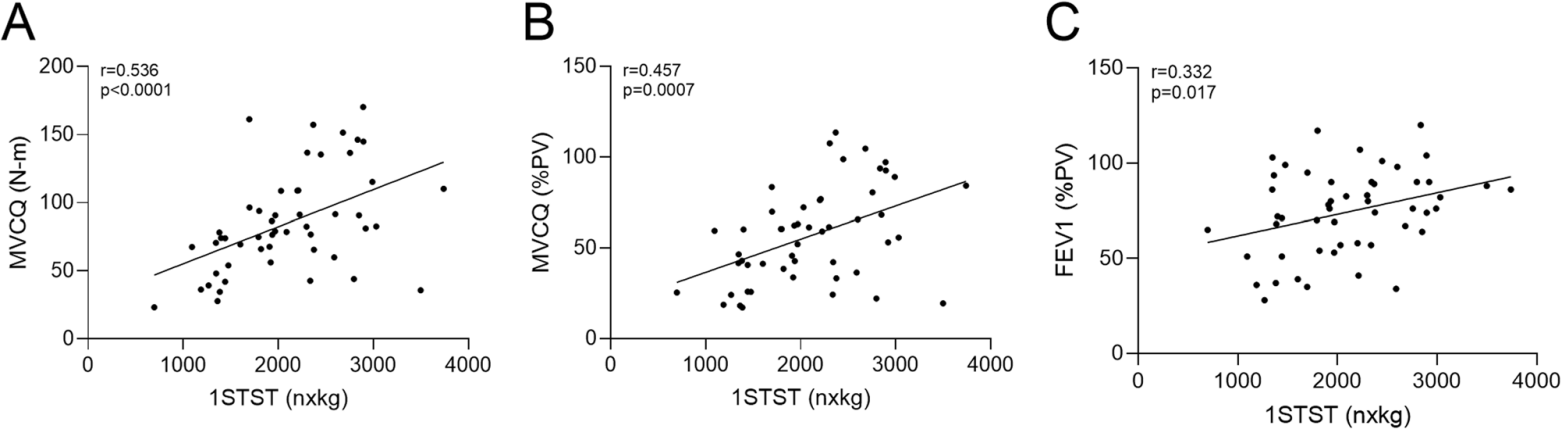
Correlation between the 1STST and FEV1 or MVCQ in the stable CF population. **A** Correlation between the 1STST expressed as a product of the bodyweight and the MVCQ expressed in Newton-meter, in the stable CF population. **B** Correlation between the 1STST expressed as a product of the bodyweight and the MVCQ expressed in percentage of the predicted values, in the stable CF population. **C** Correlation between the 1STST expressed as a product of the bodyweight and the FEV1 expressed in percentage of the predicted values, in the stable CF population. Correlation values were determined by Pearson test if parametric (**B**, **C**) and by Spearman test if non-parametric data (**A**). N = 51. *Definition of abbreviations*: 1STST, one-minute sit-to-stand test; nxkg, number of repetitions as a product of bodyweight expressed in kilogram; MVCQ, maximal isometric voluntary contraction of the quadriceps; N-m Newton-meter; PV, predicted values; FEV1, forced expiratory volume in one second; CF, cystic fibrosis; N, number of patients

The 1STST was reduced (79% (7; 142—min–max) of the predicted values (%PV)) as well as the MVCQ to 57%PV (26—SD). A moderate correlation between the 1STST expressed in number of repetitions as a product of the bodyweight and the MVCQ was observed (Figs. 1A, B). Moreover, we found a moderate correlation between the 1STST and the severity of the disease assessed by the forced expiratory volume in one second (FEV1) (Fig. 1C). No correlation was found between the 1STST expressed in number of repetitions and the MVCQ in N-m (r = 0.167). Except for the MVCQ which was significantly lower in *Pseudomonas aeruginosa* chronically infected patients (Psa ( +): MVCQ 69.57 N-m (34.70; 161.30), Psa (-): MVCQ 90.74 N-m (23.22; 170.30), p = 0.03) and Psa ( +): MVCQ 44.6%PV (21.12), Psa (-): MVCQ 63.94%PV (26.56), p = 0.008), we did not observe any other differences in 1STST and MVCQ regarding to the genotype, the pancreatic status or the patient’s microbiology (Additional file 1: Figure S1).

### Peripheral muscular strength and functional exercise capacity in children and adult patients with CF treated with IV antibiotherapy

The average duration of the IV antibiotherapy was 16.2 days. The initial 1STST was decreased (69%PV (41; 126)) as well as the MVCQ (50%PV (24; 142)). Thirty-six percent of the patients presented an acute exacerbation at the beginning of the IV antibiotherapy and fifty-three percent of the patients were hospitalized according to the criteria previously described in the methods. This treated population was rather moderately active with a mean number of steps per day of 8371/24 h (4464).

**Fig. 2:**
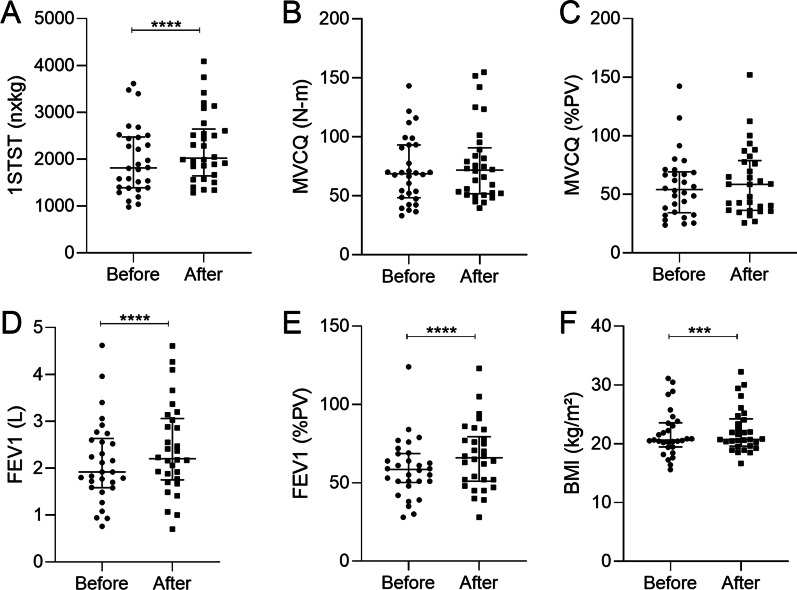
Comparison of the clinical characteristics before and after IV antibiotic treatment. **A** 1STST expressed as a product of the bodyweight before and after antibiotic treatment. **B** MVCQ expressed in Newton-meter before and after antibiotic treatment. **C** MVCQ expressed in percentage of the predicted values before and after antibiotic treatment. **D** FEV1 expressed in liter before and after antibiotic treatment. **E** FEV1 expressed in percentage of the predicted values before and after antibiotic treatment. **F** BMI expressed in body mass divided by the square of body height before and after antibiotic treatment. p-values were determined by Wilcoxon test. Bars indicate median and interquartile ranges. N = 51. *, *p* ≤ 0,05; **, *p* ≤ 0,01; ***, *p* ≤ 0,001; ****, *p* ≤ 0,0001; *Definition of abbreviations*: 1STST, one-minute sit-to-stand test; nxkg, number of repetitions as a product of bodyweight expressed in kilogram; MVCQ, maximal isometric voluntary contraction of the quadriceps; N-m Newton-meter; PV, predicted values; FEV1, forced expiratory volume in one second; L, liter; BMI, body mass index; kg/m2; kilogram divided by the square meter CF, cystic fibrosis; N, number of patients

The 1STST significantly improved after the antibiotic treatment (before: 1814 nxkg (978; 3612); after: 2020 nxkg (1287; 4089), p < 0.0001) (Fig. 2A). This increase is also observed when 1STST is expressed as number of repetitions or percentage of predicted values (Additional file 1: Figure S2). No correlation between the gain of 1STST (expressed as number of repetitions or percentage of predicted values) and the BMI was present (Additional file 1: Figure S2). Thirteen patients out of the 30, showed an improvement of more than 5 repetitions after IV antibiotics which is considered as clinically relevant (Additional file 1: Figure S3) [20]. Concerning the improvement of MVCQ, only a positive trend was shown (Figs. 2B, C). The increase of 1STST was moderately correlated to MVCQ improvement (r = 0.441; p = 0.02) (not shown). As expected, after the antibiotherapy, FEV1 and BMI were enhanced (Figs. 2D–F). Of note, we did not see any correlation between the gain of the 1STST or the MVCQ and the gain of the FEV1 or physical activity of the patients. No significant difference was found regarding to the gain in the 1STST depending on the number of steps (> 5000 steps/day is considered as active and < 5000 steps/day as sedentary person).

### Comparison between the clinical characteristics before and after IV antibiotic treatment regarding to the localization (home versus hospital)

No significant difference in the severity of the disease was observed between patients treated at home or at hospital except for their age (Additional file 1: Table S1). However, hospitalized patients tended to show a more severe profile (genotype, sweet chloride, pancreatic insufficiency, *Pseudomonas aeruginosa* chronic infection, BMI, FEV1 and proportion of exacerbations).

**Fig. 3:**
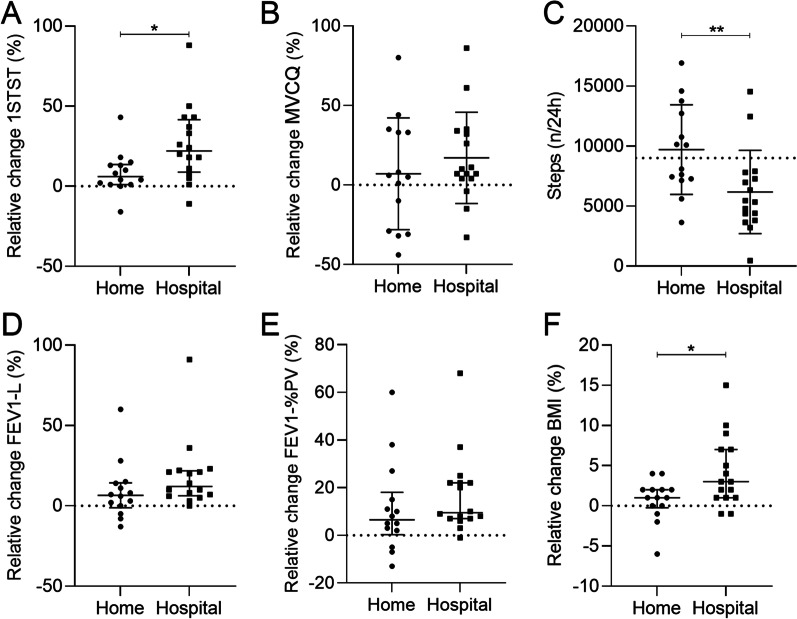
Comparison between the clinical characteristics before and after IV antibiotic treatment regarding to the localization (home versus hospital). **A** Relative change of the 1STST expressed as a product of the bodyweight, before and after IV antibiotic treatment at home and in hospital. **B** Relative change of the MVCQ expressed in Newton-meter, before and after IV antibiotic treatment at home and in hospital. **C** Average of steps per day at the beginning of the antibiotic treatment in CF patients treated at home or in hospital. **D** Relative change of the FEV1 expressed in liter, before and after IV antibiotic treatment at home and in hospital. **E** Relative change of the FEV1 expressed in percentage of the predicted values, before and after IV antibiotic treatment at home and in hospital. **F** Relative change of the BMI expressed in body mass divided by the square of body height, before and after IV antibiotic treatment at home and in hospital. P-values were determined by Mann–Whitney (**A**, **D**, **E**, **F**) test or unpaired T-test (**B**, **C**). Bars indicate means and standard deviation (**B**, **C**) data or median and interquartile ranges (**A**, **D**, **E**, **F**). N = 30. *, *p* ≤ 0.05; **, *p* ≤ 0.01; ***, *p* ≤ 0.001; ****, *p* ≤ 0.0001. *Definition of abbreviations*: 1STST, one-minute sit-to-stand test; MVCQ, maximal isometric voluntary contraction of the quadriceps; PV, predicted values; FEV1, forced expiratory volume in one second; L, liter; BMI, body mass index; CF, cystic fibrosis; N, number of patients

Both hospitalized and home treated patients significantly improved after the IV antibiotherapy regarding to the 1STST (nxkg). We found a significant greater improvement in the 1STST in hospitalized patients (22% (− 11; 88) *versus* 6% (− 16; 43), p = 0.01) compared to patients treated at home whereas no significant change in the MVCQ was shown 17% (28.64) versus 7% (35.11), p = 0.4) (Fig. 3A, B). Physical activity of hospitalized patient was significantly lower than patients treated at home (Fig. 3C). The mean number of steps per day was lower in hospital than at home, 6175/24 h (3465) versus 9702/24 h (3732), p = 0.006) (Fig. 3C). No significant differences were assessed regarding to FEV1 or BMI improvement according to the localisation of the IV antibiotherapy (Fig. 3D–F).

### Comparison between clinical characteristics before and after IV antibiotic treatment regarding to the indication (elective versus exacerbation)

**Fig. 4:**
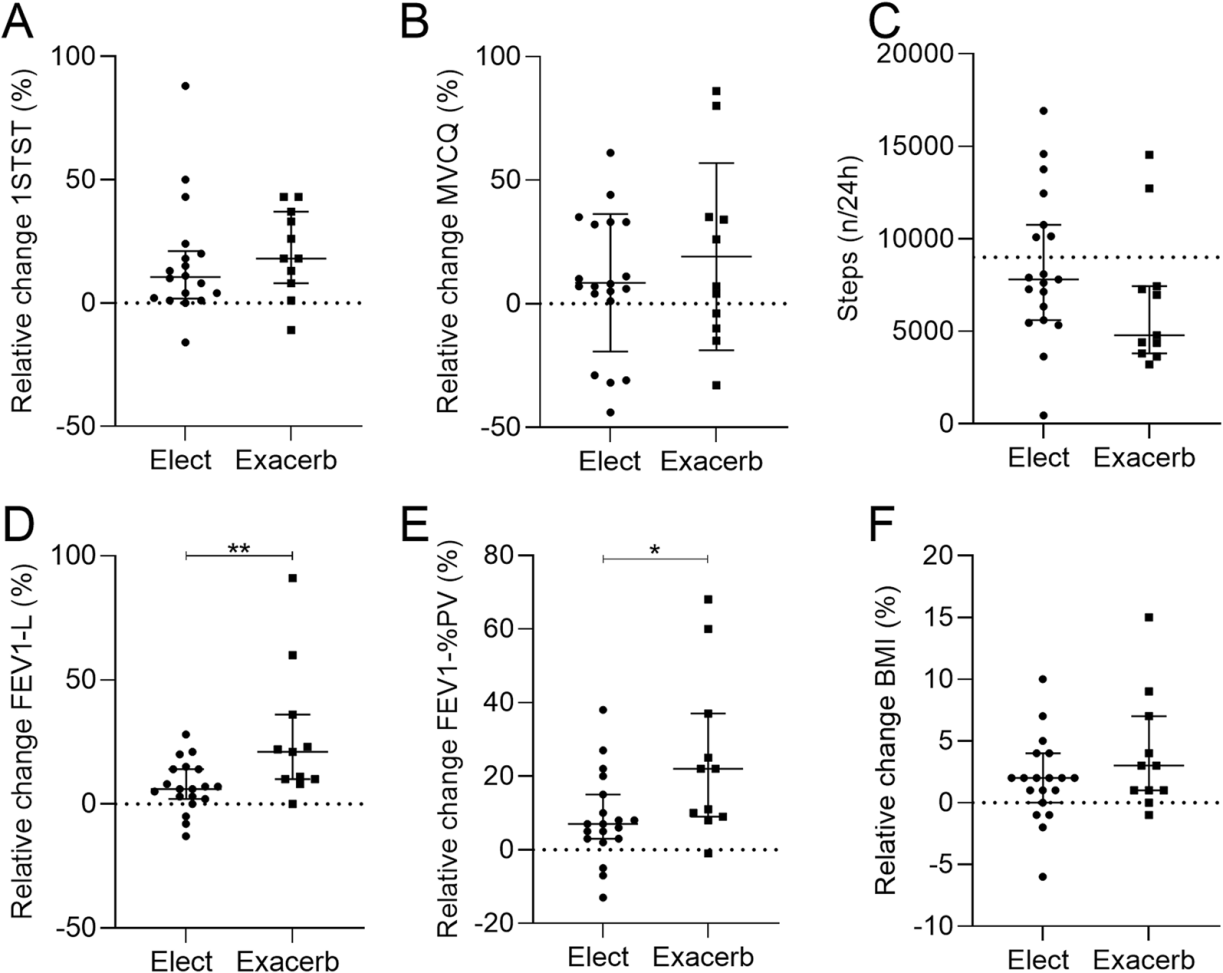
Comparison between clinical characteristics before and after IV antibiotic treatment regarding to the indication (elective versus exacerbation). **A** Relative change of the 1STST expressed as a product of the bodyweight, before and after IV antibiotic treatment at home and in hospital. **B** Relative change of the MVCQ expressed in Newton-meter, before and after IV antibiotic treatment at home and in hospital. **C** Average of steps per day at the beginning of the antibiotic treatment in patients with CF treated at home or in hospital. **D** Relative change of the FEV1 expressed in liter, before and after IV antibiotic treatment at home and in hospital. **E** Relative change of the FEV1 expressed in percentage of the predicted values, before and after IV antibiotic treatment at home and in hospital. **F** Relative change of the BMI expressed in body mass divided by the square of body height, before and after IV antibiotic treatment at home and in hospital. P-values were determined by Mann–Whitney test (**A**, **C**, **D**, **E**, **F**) test or unpaired T-test (**B**). Bars indicate means and standard deviation if parametric data (**B**) or median and interquartile ranges (**A**, **C**, **D**, **E**, **F**). N = 30. *, *p* ≤ 0,05; **, *p* ≤ 0,01; ***, *p* ≤ 0,001; ****, *p* ≤ 0,0001. *Definition of abbreviations*: 1STST, one-minute sit-to-stand test; MVCQ, maximal isometric voluntary contraction of the quadriceps; PV, predicted values; FEV1, forced expiratory volume in one second; L, liter; BMI, body mass index; CF, cystic fibrosis; N, number of patients

No significant difference in age neither in the severity of the disease was observed between elective or acute IV antibiotherapy (Additional file 1: Table S2). However, exacerbated patients tended to show a lower lung function and a higher inflammatory profile at admission than elective patients and they were preferentially hospitalized. The gain in 1STST or MVCQ was not higher in exacerbated patients in comparison to elective patients (Fig. 4A, B). The 1STST significantly improved in both groups after the IV antibiotherapy. Physical activity of exacerbated patient tends to be reduced in comparison to elective patients (Fig. 4C). Contrarily to BMI, a significant difference was observed in the FEV1 improvement according to the indication of the IV antibiotherapy (Fig. 4D–F).

## Discussion

This study showed a dramatic reduction of the MVCQ assessed by the dynamometer in the stable patients with CF in comparison to the general population [3]. The 1STST was also decreased in the stable patients with CF in comparison to the general population (79%PV). Overall, we found similar values assessing the 1STST in patients with CF when as percentage of predicted values (79%PV) than in other studies (71%PV and 75%PV, respectively) [21, 22]. Of note, our sample was the only one including children and was larger. In patients with COPD, 1STST is estimated around 50%PV [18]. Obviously, COPD patients are older than CF patients and a decline in performance in absolute values across age groups has been described, like in CF [26].

Most importantly, the 1STST seems to be a good alternative to the dynamometer in order to assess the quadriceps force in patients with CF. Indeed, we showed a positive correlation between both tests. This is supported by the fact that our correlation coefficients are either similar to previous studies conducted in CF (r = 0.520; p = 0.008), or better than in COPD (r = 0.424, p = 0.03) [22, 29]. In addition, the 1STST has the advantage over the dynamometer, to assess functional exercise capacity of the patients. Moreover, the 1STST showed moderate correlation values with the disease severity, expressed by the FEV1. This was also previously described in CF and in COPD patients [21, 30].

We showed a reduced 1STST and MVCQ assessed by the dynamometer at the beginning of the IV antibiotherapy in comparison to the general population (69%PV and 50%PV respectively). Similarly, Wiebolt et al. showed an 8% reduction of MVCQ assessed by the dynamometer for the same patient before and 1 month after the IV antibiotherapy [10]. In contrast, Burtin et al. did not show any significant differences at the admission, using both a voluntary and an involuntary method to assess the quadriceps force (femoral nerve stimulation) [12]. Nevertheless, by the fact that at the beginning of an exacerbation patients are often more symptomatic, a voluntary test could underestimate the muscular force compared to an involuntary test. In parallel, COPD patients had a higher reduction of the quadriceps force when registered at the admission of an exacerbation (22%) [31].

After the IV antibiotherapy, we assessed for the first time in CF a significant gain in the 1STST, irrespective of the localisation or of the indication of the antibiotherapy. This could partly due to the rehabilitation program in hospital as we observed that the gain of 1STST was higher when the antibiotherapy was performed in the hospital compared to home (Fig. 3A) [20, 21]. A learning effect cannot be excluded totally, even if the subject trained partly before. Furthermore, 13 patients (43%) showed a clinically relevant improvement in the number of repetitions as it was higher than the minimal clinically important difference (5 repetitions) [21]. In COPD, it has been previously shown that the 1STST was sensitive to a rehabilitation program but did not seem correlated to the exacerbation rate [18, 30]. Regarding to the MVCQ, we showed only a trend toward an improvement after the IV antibiotherapy irrespective of the localisation or the indication of the IV antibiotherapy. Wiebolt et al. described a significant enhancement after the IV antibiotherapy given for an exacerbation in patients with CF and conducted at the hospital without any specific rehabilitation program; Selvadurai et al. showed also a significant improvement of the quadriceps force, in children, but here, only with resistance exercise program [10, 11]. In COPD, Spruit et al. described a 5% decline of quadriceps force after an hospitalization for an exacerbation without pulmonary rehabilitation [31].

As hypothesized, the gain in 1STST was positively and moderately correlated to the improvement of the MVCQ. However, the enhancement of the 1STST and MVCQ was not correlated to physical activity at the beginning of the IV antibiotherapy. Indeed, no significant difference was found regarding to the gain in the 1STST depending on the number of steps. We could have expected that the more active patients could have a better 1STST and quadriceps force at the admission than the sedentary patients, and would thus less ameliorate their tests after the IV antibiotherapy, as previously suggested by Burtin and Trooster [3, 12]. Thus, this discrepancy may be explained by the relatively active patients included in our study (with a mean > 8000 steps/day). A longer time of accelerometer’s wearing could have maybe be proposed [3, 10].

As Burtin, we did not see any correlation between the enhancement of the 1STST and the quadriceps force and inflammation [12]. The enhancement of the 1STST was significantly better when the IV antibiotherapy was realized at hospital, despite a significant lower level of physical activity (number of steps/24 h) due to the patient’s confinement into their room. This supports that specific rehabilitation program during the IV antibiotherapy is necessary and efficient to counteract isolation measures.

The limitations of our study are the absence of individual values (at steady state) for each patient treated by antibiotics, the lack of the evaluation of physical activity intensity and the short duration of the actimeter measurement. Only 2 studies have calculated reference values for the 1STST in the general population: Strassman et al. for people aged from 20 to 79 years old and Reychler et al. for children aged from 6 to 12 years old [32]. Between those ages (1–5 and 13–19 years old), we do not have reference values. Reference values form Strassmann et al., were used in this study. So, we extrapolated these reference values for 13 patients (aged from 9 to 19 years) which could have led to a small overestimation of their reference values. Finally, no practice 1STST was realized in our study. However, to reduce the learning effect, the test was first demonstrated by the operator, and subjects had the opportunity to perform a few practice cycles to ensure correct realization [21]. Accelerometers were used during only 3 days to avoid extra visits to the centre and the influence of the different behaviour related to the weekend although this duration could be debated.

In conclusion, as muscular strength and exercise capacity are negative prognosis factors for CF, they should be part of the routine evaluation through 1STST. The 1STST is useful to detect and follow muscular weakness during an IV antibiotherapy, in parallel with a specific rehabilitation program.

## Supplementary Information

**Additional file 1.** Supplementary figures and tables.

## Data Availability

The datasets of the current study are available from the corresponding author on reasonable request.
