# Supplementary material for: One-minute sit-to-stand test is practical to assess and follow the muscle weakness in cystic fibrosis
Source: Respir Res. 2022 Sep 23;23:266. doi: 10.1186/s12931-022-02176-6 (PMC9508743; doi:10.1186/s12931-022-02176-6)
Supplement: Supplementary file 1 — Additional file 1. Supplementary figures and tables. [file 12931_2022_2176_MOESM1_ESM.doc]

ONE-MINUTE SIT-To-STAnd test IS PRACTICAL TO ASSESS AND FOLLOW the MUSCLE WEAKNESS in cystic fibrosis.

**Supplementary figures**


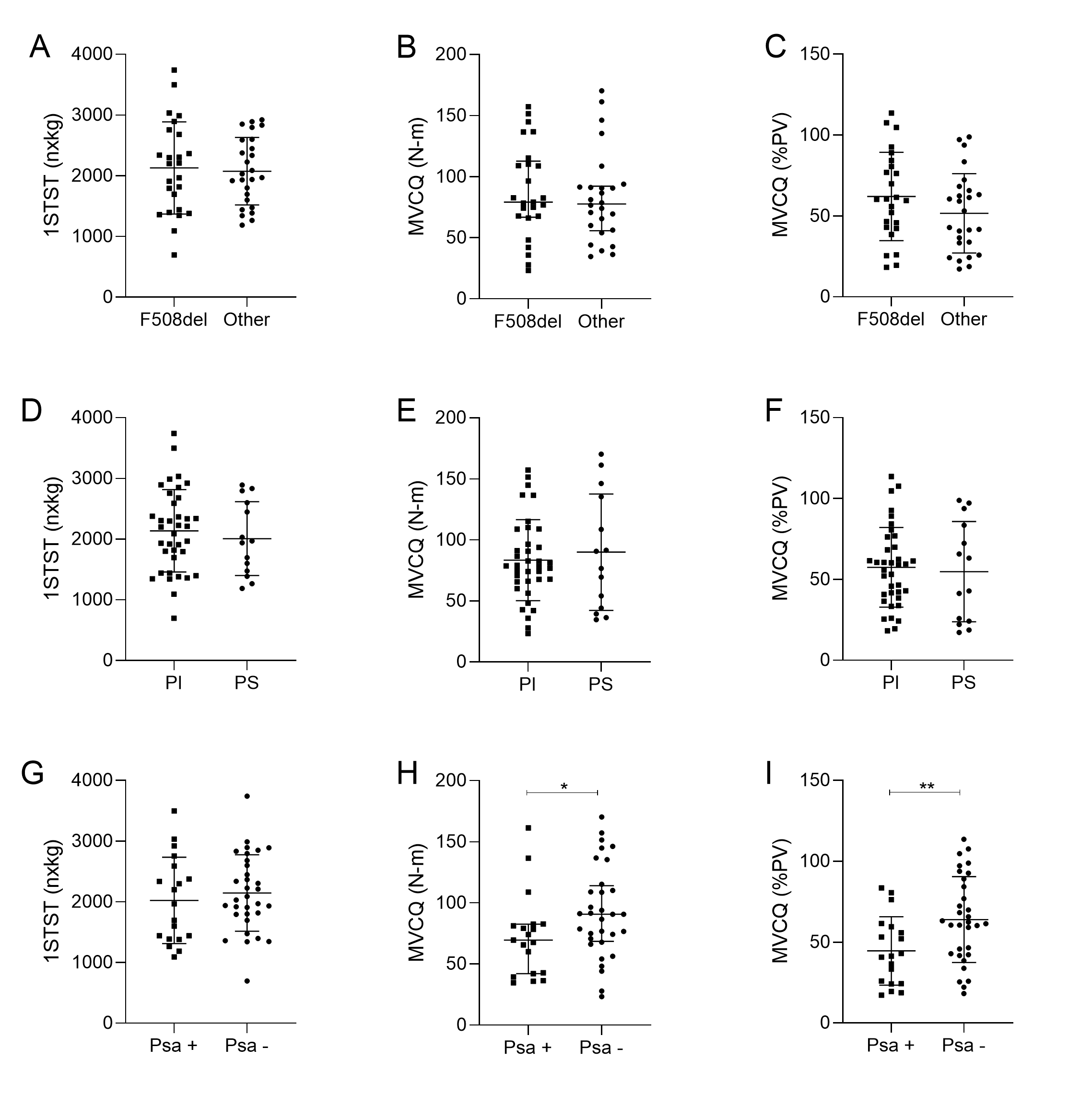


**Figure S1. Comparison of the stable CF patient’s characteristics regarding to their genotype, pancreatic function and *Pseudomonas aeruginosa* chronic infection**. (A) 1STST expressed as a product of the bodyweight in F508del homozygous patients and in patients with other CF mutations. (B) MVCQ expressed in Newton-meter in F508del homozygous patients and in patients with other CF mutations. (C) MVCQ expressed in percentage of the predicted values in F508del homozygous patients and in patients with other CF mutations. (D) 1STST expressed as a product of the bodyweight in pancreatic insufficient and pancreatic sufficient patients. (E) MVCQ expressed in Newton-meter in pancreatic insufficient and pancreatic sufficient patients. (F) MVCQ expressed in percentage of the predicted values in pancreatic insufficient and pancreatic sufficient patients. (G) 1STST expressed as a product of the bodyweight in patients chronically infected by *Pseudomonas aeruginosa* or not. (H) MVCQ expressed in Newton-meter in patients colonised by *Pseudomonas aeruginosa* or not. (I) MVCQ expressed in percentage of the predicted values in patients chronically infected by *Pseudomonas aeruginosa* or not. p-values were determined by Mann-Whitney test (B, H) and a Student t-test (A, C, D, E, F, G, I). Bars indicate means and standard deviation (A, C, D, E, F, G, I) or median and interquartile ranges (B,H). N=51. *, *p*≤0,05; **, *p*≤0,01; ***, *p*≤0,001; ****, *p*≤0,0001; CF, cystic fibrosis; 1STST, one-minute sit-to-stand test; n x kg, number of repetitions as a product of bodyweight expressed in kilogram; MVCQ, maximal isometric voluntary contraction of the quadriceps; N-m Newton-meter; PV predicted values; PI, pancreatic insufficiency; PS, pancreatic sufficiency; Psa +, *Pseudomonas aeruginosa* chronic infection; Psa -, *Pseudomonas aeruginosa* free/intermittent.


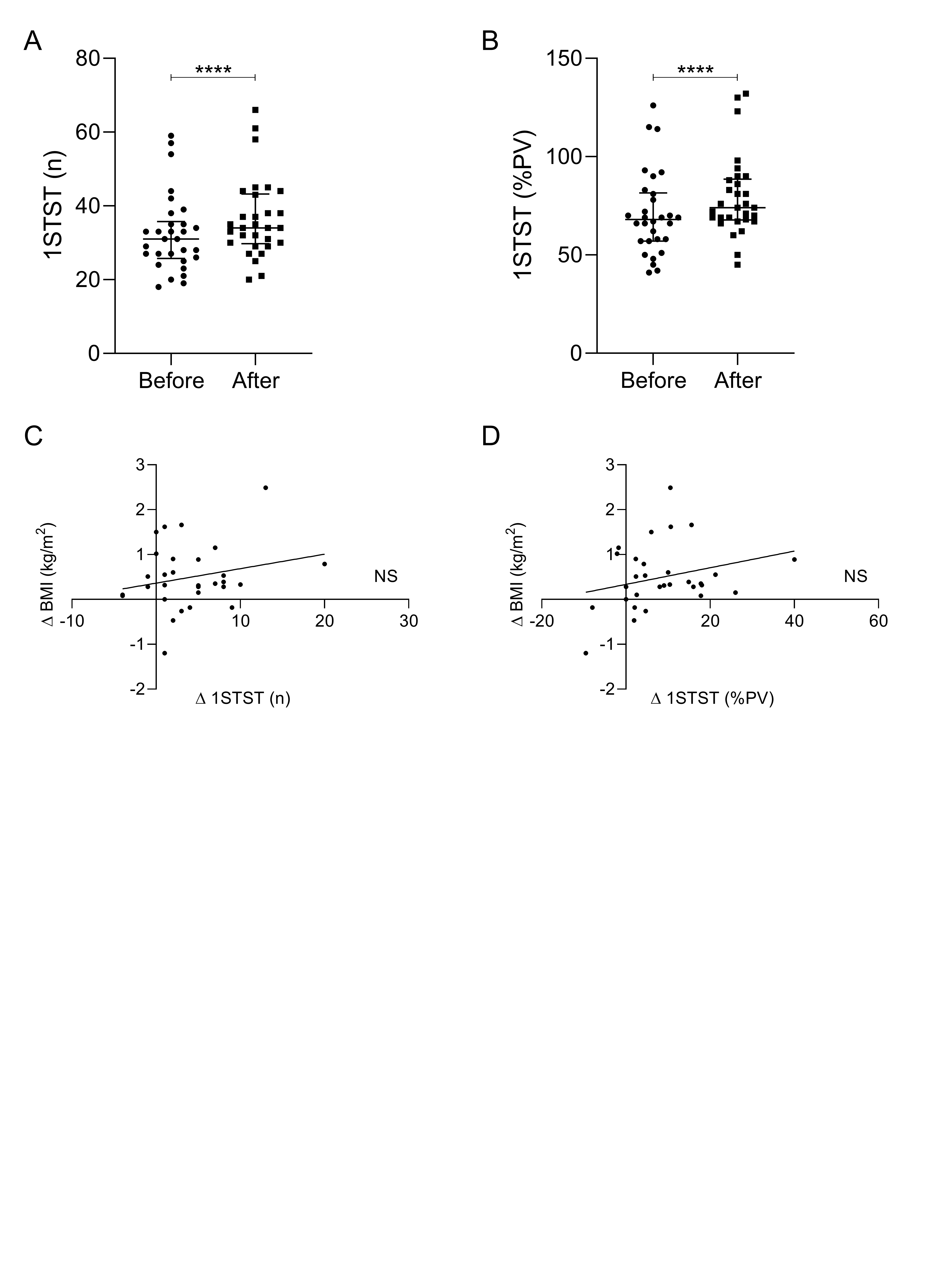


**Figure S2:** **Comparison of the 1STST and BMI before and after IV antibiotic treatment**. (A) 1STST expressed as the number of repetitions before and after antibiotic treatment. (B) 1STST expressed as the percentage of predicted values before and after antibiotic treatment. (C) Correlation between the BMI gain expressed in kg/m^2^ and the 1STST gain expressed in number of repetitions. (D) Correlation between the BMI gain expressed in kg/m^2^ and the 1STST gain expressed in percentage of predicted values. p-values were determined by Wilcoxon test (A, B) and by Pearson correlation. Bars indicate median and interquartile ranges. N=30. ****, *p*≤0,0001; *Definition of abbreviations*: 1STST, one-minute sit-to-stand test; PV, predicted values; BMI, body mass index; kg/m2; kilogram divided by the square meter; N, number of patients, NS, not significant.


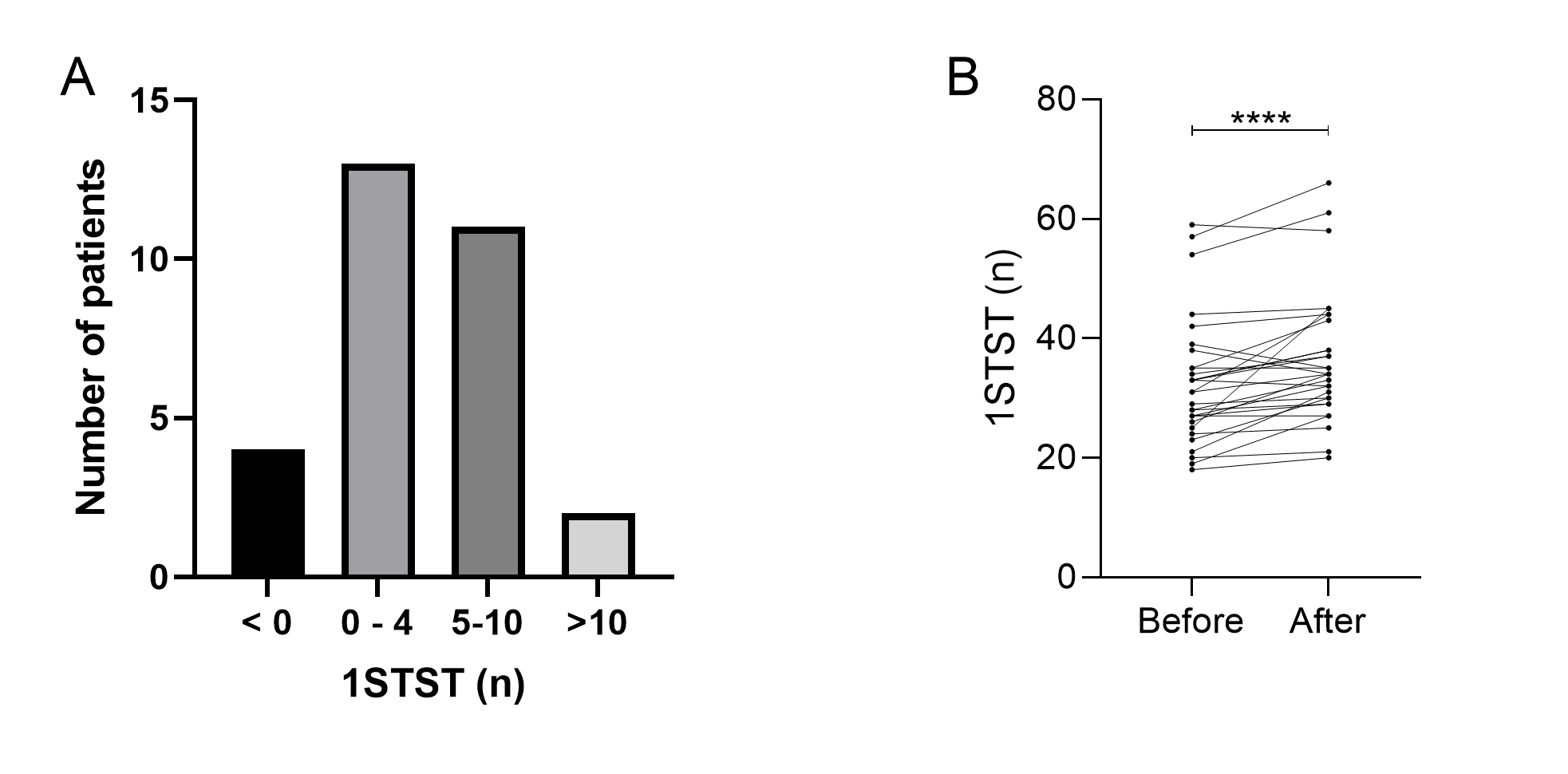


**Figure S3:** **1STST improvement after IV antibiotic treatment**. (A) The 1STST gain expressed in number of repetitions after IV antibiotherapy. (B) The evolution of the 1STST expressed in number of repetitions, for each patient, before and after IV antibiotherapy. p-values were determined by Wilcoxon test. N=30. ****, *p*≤0,0001; *Definition of abbreviations*: 1STST, one-minute sit-to-stand test; n, number of repetitions; N, number of patients

**Supplementary tables**

**Table S1**. **Comparison between the clinical characteristics at baseline regarding to the localization (home versus hospital).**

|  | **Home** | **Hospital** | ***p* value** |
| --- | --- | --- | --- |
| **Subjects, n** | 14 | 16 |  |
| **Sex (F/M)** | 8/6 | 4/12 | 0.135 |
| **Age, yrs** | 40 | 27 | 0.008 |
| **F508del/F508del (yes/no)** | 5/9 | 9/7 | 0.299 |
| **Sweat Chloride, mmol/L** | 86.98 | 102.68 | 0.193 |
| **Pancreatic sufficiency (yes/no)** | 7/7 | 3/13 | 0.122 |
| ***Pseudomonas aeruginosa* chronic infection (yes/no)** | 10/4 | 5/11 | 0.660 |
| **Diabetes (yes/no)** | 3/11 | 3/13 | 1 |
| **Smokers (yes/no)** | 0/14 | 3/13 | 0.228 |
| **BMI, kg/m²** | 23.41 | 20.31 | 0.850 |
| **FEV1, L** | 1.93 | 2.32 | 0.24 |
| **FEV1, % PV** | 54.14 | 62.94 | 0.257 |
| **1STST, n** | 34 | 31 | 0.525 |
| **1STST, %PV** (1) | 76.74 | 65.81 | 0.770 |
| **MVCQ, N-m** | 82.16 | 62.23 | 0.12 |
| **MVCQ, %PV** (2) | 63.34 | 51.54 | 0.498 |
| **Exacerbation (yes/no)** | 3/11 | 8/8 | 0.812 |
| **CRP, mg/L** | 7.88 (n=13) | 15.01 | 0.812 |
| **IgG, g/L** | 12.02 (n=13) | 12.48 (n=14) | 0.375 |
| **Chest X-Ray – new infiltrate (yes/no)** | 0/4 (n=4) | 4/5 (n=9) | 0.228 |

Data are means. Chi-Square test and Mann-Withney test are used to determine the *p* value. N is specified when data are missing. *Definition of abbreviations*: n, number; F, female; M, male; yrs, years; mmol/L, millimoles per liter; BMI, body mass index; kg/m2; kilogram divided by the square meter; FEV1, forced expiratory volume in one second; L, liter; PV, predicted values; 1STST, the one-minute sit-to-stand test; MVCQ, maximal isometric voluntary contraction of the quadriceps; N-m, Newton-meter; CRP, C-reactive protein; mg/L, milligram per liter; IgG, G-immunoglobulin; g/L, gram per liter.

**Table S2**. **Comparison between clinical characteristics at baseline regarding the indication (elective versus exacerbation).**

|  | **Elective** | **Exacerbation** | ***p* value** |
| --- | --- | --- | --- |
| **Subjects, n** | 19 | 11 |  |
| **Sex (F/M)** | 9/10 | 3/8 | 0.442 |
| **Age, yrs** | 36 | 27 | 0.103 |
| **F508del/F508del (yes/no)** | 11/8 | 3/8 | 0.142 |
| **Sweat Chloride, mmol/L** | 94.68 | 96.50 | 0.703 |
| **Pancreatic sufficiency (yes/no)** | 6/13 | 4/7 | 1 |
| ***Pseudomonas aeruginosa* chronic infection (yes/no)** | 11/8 | 4/7 | 0.450 |
| **Diabetes (yes/no)** | 4/15 | 2/9 | 1 |
| **Smokers (yes/no)** | 2/17 | 1/10 | 1 |
| **BMI, kg/m²** | 21.72 | 21.82 | 1 |
| **FEV1, L** | 1.92 | 2.51 | 0.123 |
| **FEV1, %PV** | 56.37 | 63.09 | 0.672 |
| **1STST, n** | 34 | 29 | 0.417 |
| **1STST, %PV** (1) | 75.96 | 61.99 | 0.085 |
| **MVCQ, N-m** | 82.16 | 62.23 | 0.703 |
| **MVCQ, %VP** (2) | 58.21 | 54.96 | 0.832 |
| **Hospitalization (yes/no)** | 8/11 | 8/3 | 0.142 |
| **CRP, mg/L** | 4.72 (n=18) | 23.42 | 0.084 |
| **IgG, g/L** | 11.25 (n=18) | 14.26 (n=9) | 0.668 |
| **Chest X-Ray – new infiltrate (yes/no)** | 1/3 (n=4) | 3/6 (n=9) | 1 |

Data are means. Chi-Square test and Mann-Withney test are used to determine the *p* value. N is specified when data are missing. *Definition of abbreviations*: n, number; F, female; M, male; yrs, years; mmol/L, millimoles per liter; BMI, body mass index; kg/m2; kilogram divided by the square meter; FEV1, forced expiratory volume in one second; L, liter; PV, predicted values; 1STST, the one-minute-sit-to-stand test; MVCQ, maximal isometric voluntary contraction of the quadriceps; N-m, Newton-meter; CRP, C-reactive protein; mg/L, milligram per liter; IgG, G-immunoglobulin; g/L, gram per liter.

**Supplementary references**

1. Strassmann A, Steurer-Stey C, Lana KD, Zoller M, Turk AJ, Suter P, et al. Population-based reference values for the 1-min sit-to-stand test. Int J Public Health [Internet]. 2013 Dec 24;58(6):949–53. Available from: http://link.springer.com/10.1007/s00038-013-0504-z

2. Hogrel J-Y, Payan CA, Ollivier G, Tanant V, Attarian S, Couillandre A, et al. Development of a French Isometric Strength Normative Database for Adults Using Quantitative Muscle Testing. Arch Phys Med Rehabil [Internet]. 2007 Oct;88(10):1289–97. Available from: http://linkinghub.elsevier.com/retrieve/pii/S0003999307012828
